# Complementary and Alternative Medicine-Induced Combined Hepatorenal Toxicity: A Case Report

**DOI:** 10.7759/cureus.100197

**Published:** 2025-12-27

**Authors:** George S Zacharia, Vedashree Ravte, Abhilasha Jyala, Elona Shehi

**Affiliations:** 1 Internal Medicine, BronxCare Health System, New York, USA; 2 Gastroenterology, BronxCare Health System, New York, USA

**Keywords:** complementary and alternative medicine, drug-induced liver injury (dili), hepatotoxicity, liver function test (lft), rucam score

## Abstract

Drug-induced liver injury (DILI) is a leading cause of liver disease globally, with complementary and alternative medicine (CAM) representing a significant contributor due to its widespread use and limited regulation. CAM encompasses diverse practices and products, with regional and ethnic diversity, many of which are used to treat liver disease. Even though a few natural remedies have been utilized for centuries, most lack scientific evidence for their safety and efficacy. This report describes a 42-year-old male who self-treated his fatty liver with CAM, culminating in severe concomitant hepatic and renal dysfunctions. Comprehensive evaluation excluded viral, autoimmune, metabolic liver diseases, and obstructive biliary diseases. The Roussel Uclaf Causality Assessment Method (RUCAM) score indicated a probable causal link between CAM and DILI of hepatocellular pattern. The constituents of the CAM, extracts of *Carica papaya*, *Alstonia boonei*, and *Tetrapleura tetraptera*, have been implicated in hepatotoxicity in animal models. Discontinuing the supplements and supportive care, including N-acetyl cysteine, led to symptomatic and biochemical improvement. This case highlights the importance of clinicians' awareness of CAM-related DILI, emphasizing the need for vigilance and patient education about the potential risks associated with herbal products.

## Introduction

Complementary and alternative medicine (CAM) encompasses a broad spectrum of healthcare practices and products that typically do not follow the principles of modern evidence-based medicine. CAM tends to be based on traditional medical practices unique to each population: Ayurveda and Siddha in India, Kampo in Japan, Mapuche in Chile, Curanderismo in Mexico, Unani from the Middle East, Koryo in Korea, Chinese herbal medicine, and African and Brazilian traditional medicine. Interestingly, most of these practices rely on natural products, manual techniques, and spiritual or indigenous beliefs. At least a handful of these traditional medicines have later evolved into groundbreaking medications in modern medicine, such as artesunate from Artemisia annua, used in Chinese herbal medicine [1]. The regional beliefs, the propaganda about the safety of natural products, and falsified advertisements, combined with the gaps and surging costs in modern medicine, have always provided ample room for CAM to thrive and flourish. Drug-induced liver Injury (DILI) ranks high on the list of hepatic diseases, and CAM is implicated in hepatotoxicity in 9.8% to 76% of such cases [2,3]. CAM hepatotoxicity may be related to the active ingredients, adulterants, or contaminants in the medicinal preparation. 

## Case presentation

A 42-year-old African-American male presented with nausea, vomiting, and upper abdominal pain of three days' duration. He endorsed no altered bowel habits or overt gastrointestinal bleeding. He reported using three herbal medications for fatty liver for a couple of months; constituents of the same are reported in Table 1. He consumes alcohol intermittently but denies the use of illicit drugs and other medications. He had traveled to Africa four months before the onset of symptoms. He denied having any close contact with jaundiced individuals; no dental work, no transfusions, or surgeries. Physical examination revealed icterus but was otherwise unremarkable. The patient had no encephalopathy and was alert and oriented in time, place, person, and situation.

**Table 1:** Complimentary medications used by the patient Constituents as reported on the Internet. The authors have not analyzed the chemical composition of the herbal preparations.

Product	Components
Product A	Carica papaya, Alstonia boonei, Tetrapleura tertraptera
Product B	Carica papaya, Azadirachta indica, Spondias mombin, Veronia amygdalina, Persea americana, Mangifera indica, Ocimum viride
Product C	Naartjie, cinnamon, thyme, chamomile extract, neutral spirit

**Figure 1:**
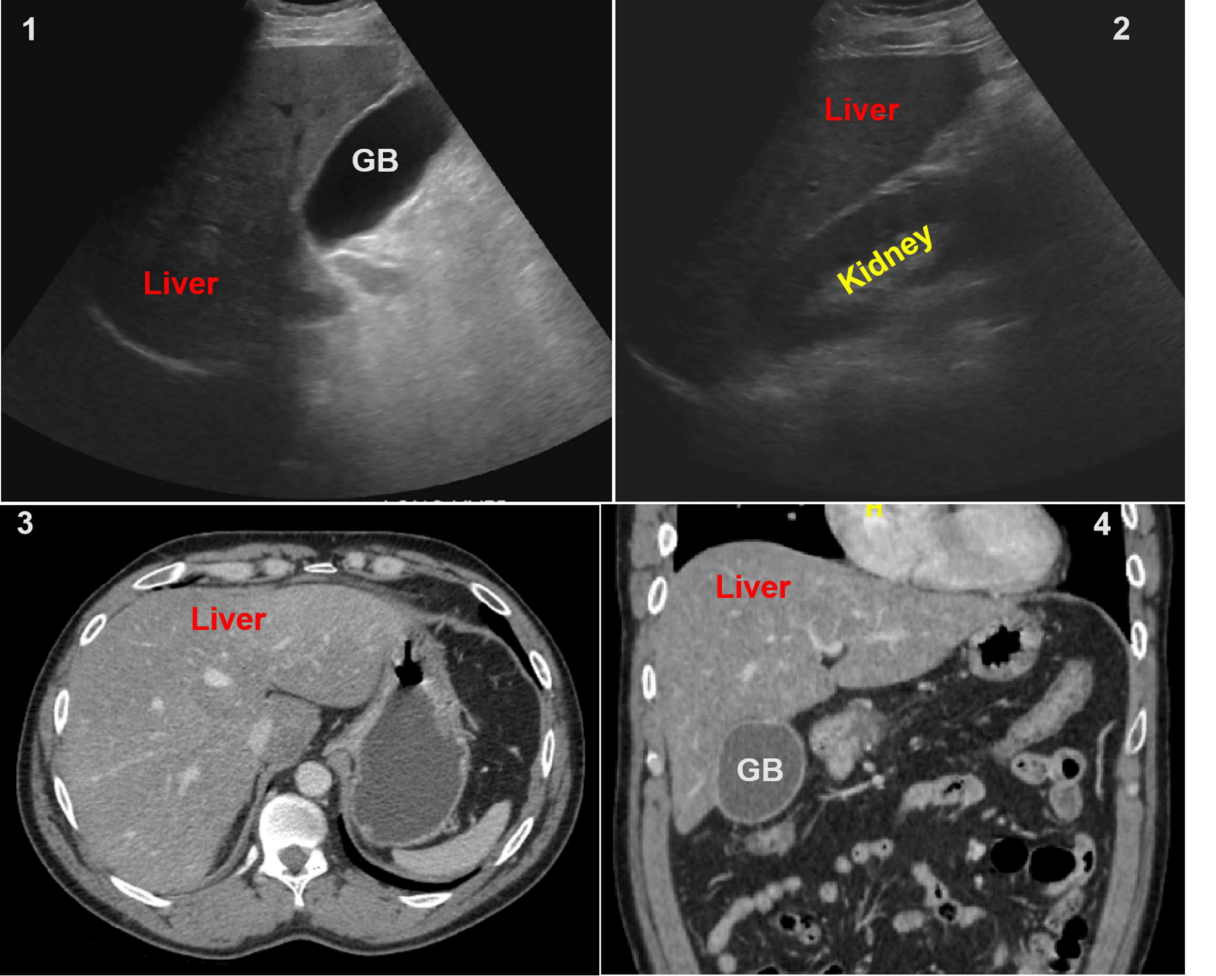
Ultrasound and computed tomography images Ultrasound images (A, B) revealed a hyperechoic liver and a distended gallbladder (GB) with borderline wall thickness, but without cholelithiasis. Computed tomography (C, D) revealed diffuse low-attenuation of the liver compatible with fatty infiltration and a distended GB.

The hemogram was normal except for mild leukocytosis. At admission, liver chemistries revealed conjugated hyperbilirubinemia, with a total bilirubin of 3.4 mg/dL and a direct fraction of 2.3 mg/dL, alanine and aspartate aminotransferases >7000 IU/ml, alkaline phosphatase (ALP) 166, and gamma-glutamyl transpeptidase (GGT) 310, and normal protein and albumin levels. He also had acute kidney injury at admission with a blood urea nitrogen (BUN) of 25 mg/dL and creatinine of 5.8 mg/dL. He was non-oliguric, with apparently normal urinalysis. The fractional excretion of sodium (FeNa) was 2.8, consistent with intrinsic renal failure. The serum ethanol presentation was 40mg/dL, while serum acetaminophen and acetylsalicylic acid levels were undetectable. The urine toxicology screen was reported negative as well. Viral markers for hepatitis A (HAV), hepatitis B (HBV), hepatitis C (HCV), hepatitis E (HEV), herpes simplex and zoster, cytomegalovirus, and Epstein-Barr viruses were reported to be negative for active infection. Serum ceruloplasmin was 15 mg/dL, while the 24-hour urine copper excretion was normal. Serum transferrin saturation was <45%, while alpha-1 antitrypsin level was within the normal range. Evaluation for autoimmune liver disease, including antinuclear (ANA), smooth muscle (ASMA), liver-kidney-microsomal (ALKM), and mitochondrial antibodies (AMA), was non-contributory. An overview of the biochemical and etiological workups is tabulated in Tables 2-3, respectively. The abdominal ultrasound (USG) and computerized tomography (CT) revealed a steatotic liver with no biliary dilatation and a borderline gall bladder wall thickness of 3 mm (Figure 1). Slit lamp examination did not reveal any Kayser-Fleischer ring. 

**Table 2:** A summary of the biochemical profile ALT: alanine aminotransferase; AST: aspartate aminotransferase; ALP: alkaline phosphatase; INR: international normalized ratio; BUN: blood urea nitrogen

Biochemical parameters	6m prior	Day 0	Day 3	Day 5	Day 10	Day 90	Normal
Bilirubin, total (mg/dL)	1.2	3.4	6.1	3.4	1.8	0.3	0.2-1.1
Bilirubin, direct (mg/dL)	0.3	2.3	4.9	2	1	<0.2	0.1-0.2
ALT (IU/L)	89	>7000	2431	1049	404	87	5-40
AST (IU/L)	73	>7000	1952	272	59	66	9-36
ALP (IU/L)	65	166	121	198	151	74	42-144
INR	0.90	2.78	1.56	0.95	0.92	NA	0.9-1.1
Creatinine (mg/dL)	1	5.8	10.4	9.8	2	0.9	0.5-1.5
BUN (mg/dL)	12	25	41	59	34	16	6-20

**Table 3:** Excerpt of the etiological work-up HAV: hepatitis A virus; HBV: hepatitis B virus; HCV: hepatitis C virus; HDV: hepatitis D virus; HEV: hepatitis E virus; HSV: herpes simplex virus; EBV: Epstein-Barr virus; CMV: cytomegalovirus; ANA: anti-nuclear antibody; ALKM: anti-liver-kidney-microsomal antibody; ASMA: anti-smooth muscle antibody; AMA: anti-mitochondrial antibody; ANCA: anti-neutrophil cytoplasmic antibody

Etiologies		Results
Infections	HAV/HBV/HCV/HDV/HEV	Negative
	HSV/EBV/CMV/Leptospira	
Autoimmune	ANA/ALKM/ASMA	Negative
	AMA/ANCA	
Metabolic	Transferrin Saturation	31.5% (Normal: <45%)
	ɑ1-anti-trypsin	116 (Normal: 83-199 mg/dL)
	Ceruloplasmin	15 (Normal: 14-30 mg/dL)
	24-hour urine copper	45 (Normal: 15-60 mcg)
Drugs	Acetaminophen	< 5 (Normal: < 5 μg/mL)
	Acetylsalicylic acid	< 0.5 (Normal: < 0.5 mg/mL)
	Urine drug screen	Negative

The patient was managed with an N-acetyl cysteine (NAC) infusion: initial dose of 150 mg/kg infused over the first 60 minutes, followed by 50 mg/kg infused over the next four hours, and subsequently 100 mg/kg over 16 hours, vitamin K: intravenous, 10 mg/day for three days, and supportive measures. All alternative medications were withheld, and he was counseled against their use. The patient was recommended a liver biopsy for confirmation of diagnosis; however, he opted not to proceed with the invasive procedure. Over two weeks in the hospital, he never developed encephalopathy and continued to be non-oliguric with serial improvement in liver and kidney functions. Following discharge, with symptomatic and biochemical improvement, he was regularly followed up in the outpatient clinic, and his clinical course remained uneventful. 

## Discussion

CAM has existed for centuries, even before modern medicine and evidence-based medical practices. Scriptures suggest that these practices date back 3,000 to 5,000 years to the pre-Christian era, especially in Ayurveda and traditional Chinese medicine. Despite a lack of scientific evidence on their utility or futility, these practices are closely linked to regional and spiritual beliefs and are often religiously followed in the developing world. However, such practices are usually not legalized in most industrialized countries, though population migration results in their use in the Western world [4]. With the ever-growing burden of diseases and medications, DILI has evolved into a significant cause of liver disease. Recent literature suggests that CAM contributes significantly to DILI through its active ingredients or contaminants [2,5]. Most cases of CAM DILI follow an idiosyncratic pathophysiology and a hepatocellular injury pattern [6]. Worldwide, traditional Chinese medications are most frequently implicated in DILI. The list of implicated agents is exhaustive; often reported from Asia include *Breynia vitis-idaea, Aloe perfoliata var. vera, Lycopodium serratum, Glycyrrhiza glabra, Polygonum multiflorum Thunb, Podophyllum pleianthum, Ephedra sinica, Garcinia cambogia, *and* Piper methysticum *[2].

Our patient, with hepatic steatosis at baseline, was using three herbal preparations and alcohol when he developed the combined hepato-nephrotoxicity. The Roussel Uclaf Causality Assessment Method (RUCAM) score was 7, suggesting the probable relation between CAM and liver injury. The R ratio was >5, consistent with a hepatocellular DILI. A literature search identified 14 reported constituents in the CAM that the patient used. Among the constituents, *Carica papaya, Alstonia boonei*, and *Tetrapleura tetraptera* extracts have been linked to hepatotoxicity in animal models [7-12]. Nephrotoxicity has also been reported with *Carica papaya* and *Alstonia boonei* [13-15]. *Tetrapleura tetraptera*, in rodent studies, has demonstrated renal toxicity at doses exceeding 1000 mg/kg [16]. Extensive evaluation excluded the possibilities of infective and autoimmune hepatitis, Wilson's disease, hemochromatosis, alpha-1 antitrypsin deficiency, as well as alternative drug and toxic hepatopathies. Abdominal imaging ruled out biliary obstruction and sequelae of cholelithiasis. The patient endorsed ethanol consumption; however, ethanol-related hepatitis virtually never develops transaminitis exceeding 500 IU/ml [17]. The exclusion of alternate causes, the spatial association between CAM and liver dysfunction, together with prompt improvement following discontinuation of CAM, favors the diagnosis of CAM-related DILI in this patient. A combination of *Carica papaya, Alstonia boonei*, and *Tetrapleura tetraptera* might have resulted in combined hepato-renal failure in our patient. Interestingly, preparations containing the agents above are used in traditional medicine for liver diseases, and an idiosyncratic response may have led to hepatotoxicity. 

The diagnosis of DILI is one of exclusion, clubbed with evidence of a causal relationship between the implicated drug and hepatic dysfunction. Laboratory evaluation and imaging help exclude alternative causes, though they might not confirm the diagnosis. In most cases, liver biopsy reveals non-specific inflammatory changes, and the probability of making a definitive diagnosis of DILI is low [18]. Excluding the likes of acetaminophen and methotrexate, most DILIs are non-dose dependent and idiosyncratic. Genetic and environmental factors might play a role in DILI, e.g., amoxicillin-clavulanate and methotrexate. The RUCAM score assesses the causality with the R-ratio and classifies DILI as hepatocellular, cholestatic, or mixed [18-20]. The key to treatment is the withdrawal of the implicated drug as soon as possible. There is no definitive medical intervention in patients with DILI except for acetaminophen hepatotoxicity. The 2021 American College of Gastroenterology guidelines suggest the use of NAC in adults with early-stage acute liver failure and steroids in those with an autoimmune hepatitis-like phenotype [18]. The prognosis depends upon multiple variables; hepatocellular DILI carries a higher risk of liver failure and transplantation, while cholestatic DILI predicts a higher risk of chronic liver disease. A Model for End-Stage Liver Disease (MELD) score exceeding 19 or modified Hy’s law predicts liver-related mortality in DILI [18].

## Conclusions

Drug-induced hepatotoxicity is a leading cause of liver disease worldwide, and CAM contributes to a sizeable proportion of DILI. Medications or preparations, including those used for the treatment of liver diseases in traditional medicine, have been linked with DILI. The hepatotoxic effects of CAM are vastly under-reported and may be related to the constituents, adulterants, or pollutants in these preparations. A high index of suspicion, exclusion of alternate causes, and establishment of causality are crucial to the diagnosis, while exclusion of the potential agent(s) is vital to managing DILI.
